# Lateral armrest support improves postural alignment, respiratory mechanics, and shoulder girdle loading during seated eating in healthy young adults: a multidimensional biomechanical evaluation

**DOI:** 10.1186/s12984-026-01960-5

**Published:** 2026-05-02

**Authors:** Daisuke Tashiro, Kaito Tou, Ikoi Okano, Sayaka Nishiguchi

**Affiliations:** 1 Department of Occupational Therapy, Faculty of Rehabilitation, Kobe Gakuin University, 518 Arise Ikawadanichou, Nishi-ku, Kobe, Hyogo 651-2180 Japan; 2 Tsuji Neurology Clinic, Kobe, Hyogo Japan

**Keywords:** Postural alignment, Respiratory mechanics, Accessory respiratory muscles, Muscle stiffness, Upper-limb support device

## Abstract

**Background:**

Postural collapse and increased reliance on accessory respiratory muscles during meals can compromise ventilatory efficiency, particularly in individuals with respiratory impairment. Forward elbow-supported sitting is commonly used to unload the upper limbs; however, this posture often induces trunk flexion and cervical extension, which may adversely affect respiratory mechanics. To date, the effects of lateral armrest support on posture and respiratory function during seated eating have not been quantitatively investigated.

**Objective:**

To examine the effects of lateral armrest support on thoracic alignment, cervical posture (cervical inclination angle), shoulder muscle stiffness (upper trapezius and middle deltoid), vital capacity, and subjective comfort during seated eating in healthy young adults, compared with unsupported sitting and anterior elbow-supported sitting.

**Methods:**

Forty healthy young adults completed three randomized sitting conditions: (A) unsupported sitting, (B) anterior elbow-supported sitting, and (C) lateral armrest-supported sitting using a side-mounted armrest. Thoracic kyphosis index, cervical inclination angle, muscle stiffness of the upper trapezius and middle deltoid, vital capacity (VC), and subjective comfort were assessed. Data were analyzed using Friedman tests, with post-hoc pairwise comparisons performed using the Durbin–Conover test with Bonferroni correction.

**Results:**

Thoracic kyphosis index differed significantly across conditions (*p* = .002), with both forward elbow-supported and lateral armrest-supported sitting showing lower values than unsupported sitting. Cervical inclination angle also differed significantly (*p* < .001), with forward elbow-supported sitting demonstrating greater cervical extension than the other conditions. Upper trapezius muscle stiffness was significantly reduced in the lateral armrest-supported condition compared with unsupported and forward elbow-supported sitting (both *p* < .01). Vital capacity was significantly greater in the lateral armrest-supported condition than in unsupported sitting (*p* = .003). Subjective comfort ratings were highest in the lateral armrest-supported condition (*p* < .001).

**Conclusion:**

Lateral armrest support demonstrated biomechanical advantages in healthy young adults, including improved spinal alignment, reduced shoulder girdle loading, and greater vital capacity compared with unsupported sitting. These findings provide preliminary mechanistic insight and may inform future investigations in clinical populations; however, direct extrapolation to individuals with respiratory or neurological impairment requires further study.

## Introduction

Postural–respiratory coupling is governed by biomechanical interactions between spinal alignment, rib cage configuration, and diaphragmatic length–tension relationships. Forward trunk flexion alters thoracic geometry, reduces rib mobility, and shifts ventilatory demand toward accessory muscles of the shoulder girdle. Increased activation of these muscles, including the upper trapezius, may reflect compensatory strategies when diaphragmatic efficiency is mechanically constrained.

Eating-related seated posture represents a task-specific coordination of head, trunk, and upper-limb segments. Therefore, isolated evaluation of either spinal alignment or pulmonary function may not fully capture the integrated biomechanical demands of this activity. A multidimensional assessment incorporating spinal alignment, muscular loading, ventilatory capacity, and subjective perception provides a more comprehensive framework for understanding how upper-limb support configuration influences respiratory mechanics. Recent research has further emphasized the close interaction between posture and respiratory mechanics during functional activities.

Several biomechanical studies have demonstrated that thoracic alignment and trunk posture significantly influence chest wall movement patterns, ventilatory efficiency, and respiratory muscle recruitment during seated tasks.

These findings highlight the importance of postural configuration as a determinant of respiratory performance in daily activities [1–3].

Experimental evidence indicates that slouched sitting significantly decreases respiratory muscle strength compared with upright sitting, placing the respiratory system at a mechanical disadvantage [4]. Such postural deviations increase reliance on accessory respiratory muscles, elevate the work of breathing, and may adversely affect swallowing function by altering biomechanical alignment. Recent studies further suggest that kyphotic sitting postures can impair swallowing performance, highlighting a potential disruption of swallowing–breathing coordination [5].

Anterior elbow-supported sitting is commonly adopted during bed-based sitting, particularly in clinical and home-care settings, to unload the upper limbs and alleviate dyspnea during eating and other seated tasks. Despite its widespread use, this posture frequently induces trunk flexion and cervical extension—mechanical alterations known to reduce lung volumes and modify thoracoabdominal movement patterns [6, 7].

However, the combined postural, muscular, and respiratory consequences of anterior elbow support during eating-related activities in bed-sitting conditions have not been comprehensively quantified. Previous studies have typically examined spinal alignment, muscle activity, or pulmonary function independently. Yet, eating-related seated posture represents a coordinated biomechanical task requiring simultaneous integration of trunk alignment, shoulder girdle loading, and ventilatory mechanics. Therefore, a multidimensional assessment framework is necessary to capture the interactive effects of upper-limb support configuration on respiratory function during functional tasks.

Lateral armrest support may represent an alternative seating strategy that enables upper-limb unloading while maintaining a more upright trunk alignment in bed-sitting positions. By supporting the elbows laterally rather than anteriorly, shoulder girdle loading may be reduced without encouraging forward trunk inclination, potentially decreasing activation of accessory respiratory muscles such as the trapezius [8]. From the perspective of rehabilitation engineering, such support systems may contribute to improved ergonomic design of bed-based seating environments and enhanced respiratory efficiency during daily activities. Nevertheless, no prior studies have systematically examined how lateral armrest support influences thoracic alignment, cervical posture, accessory respiratory muscle loading, and ventilatory capacity during bed-based seated eating.

To quantify spinal alignment, the thoracic kyphosis index was selected because sagittal trunk flexion is known to influence rib cage mobility and ventilatory mechanics. Cervical inclination angle was assessed to evaluate head–trunk coordination during eating-related tasks, as cervical alignment may affect swallowing–breathing interaction and visual orientation.

Muscle stiffness of the upper trapezius was included as a non-invasive indicator of sustained shoulder girdle loading during semi-static eating posture. Increased stiffness of this muscle may reflect compensatory activation associated with altered upper-limb support and postural alignment. Previous studies have demonstrated that handheld muscle hardness measurements provide a reliable estimate of superficial muscle mechanical properties and muscular loading during postural tasks [9, 10].

Vital capacity was included to assess posture-related mechanical constraints on thoracic expansion under standardized respiratory effort. Finally, subjective comfort was evaluated to capture perceptual responses relevant to functional task tolerance.

Together, these measures provide a multidimensional assessment framework integrating spinal alignment, muscular loading, ventilatory mechanics, and perceived task demand. Therefore, the purpose of this study was to evaluate the biomechanical and respiratory effects of lateral armrest support during simulated eating in a bed-sitting position. By comparing lateral armrest-supported sitting with unsupported sitting and anterior elbow-supported sitting, this study aims to provide foundational evidence to inform the design of assistive bed-based seating systems and rehabilitation strategies.

## Method

### Study design

This study employed a repeated-measures, within-subject experimental design to examine the effects of different upper-limb support configurations on posture, muscle stiffness, respiratory function, and subjective comfort during seated eating. All participants completed three standardized seated postural conditions conducted entirely on a hospital bed:

(A) Unsupported sitting
(B) Forward elbow-supported sitting
(C) Lateral armrest-supported sitting

The order of the three conditions was counterbalanced across participants using a Latin-square approach to minimize potential order and carryover effects. All measurements were obtained within a single experimental session under controlled laboratory conditions.

### Participants

Forty healthy adults (20 males and 20 females; age range: 20–23 years) were recruited for this study. Inclusion criteria were the absence of diagnosed respiratory, neurological, or musculoskeletal disorders and the ability to maintain independent sitting. Exclusion criteria included acute respiratory infection, pain affecting sitting posture, or neurological impairment influencing postural control.

All participants provided written informed consent prior to participation. The study protocol was approved by the Ethics Committee of the Faculty of Comprehensive Rehabilitation Studies, Kobe Gakuin University (Approval No. 24 − 17, approved on 25 March 2024), and was conducted in accordance with the Declaration of Helsinki.

### Experimental setup and postural conditions

All experimental conditions were performed on a hospital bed using an overbed table (Sehonens Co., Ltd., Japan). For all three conditions, the overbed table was positioned 8 cm anterior to the abdominal surface (approximately one fist width) and adjusted to a height appropriate for simulated eating tasks.

Participants assumed a standardized eating posture across all conditions, holding chopsticks with the right hand and a dish with the left hand to replicate a realistic mealtime scenario. During the unsupported sitting condition, participants were instructed not to lean against the bed. In all three conditions, participants were instructed to lift and hold the bowl at a standardized position approximately midway between the table surface and the mouth, thereby maintaining a consistent bowl-to-mouth distance across conditions. Trunk and head positioning were visually monitored by the examiner throughout the trials to ensure adherence to the instructed posture.

Bed settings were standardized as follows:

- Backrest elevation: 65°
- Knee-gatch elevation: 15°
- No pillow use

Participants changed into loose-fitting clothing that did not restrict trunk or abdominal movement.

#### Condition A: unsupported sitting

Participants maintained an upright sitting posture with both upper limbs unsupported and lifted off the table surface. Although the overbed table was present, the arms did not contact the table, ensuring the absence of external upper-limb support.

#### Condition B: forward elbow-supported sitting

Participants placed both elbows directly on the overbed table, providing anterior upper-limb support. This configuration represented a commonly adopted forward-leaning elbow-supported posture during seated eating in clinical practice.

#### Condition C: lateral armrest-supported sitting

In this condition, bilateral lateral elbow-support components (Emi Table; Sehonens Co., Ltd., Japan) were attached to both sides of the overbed table. Participants placed both elbows on these lateral supports, providing lateral upper-limb support.

**Fig. 1:**
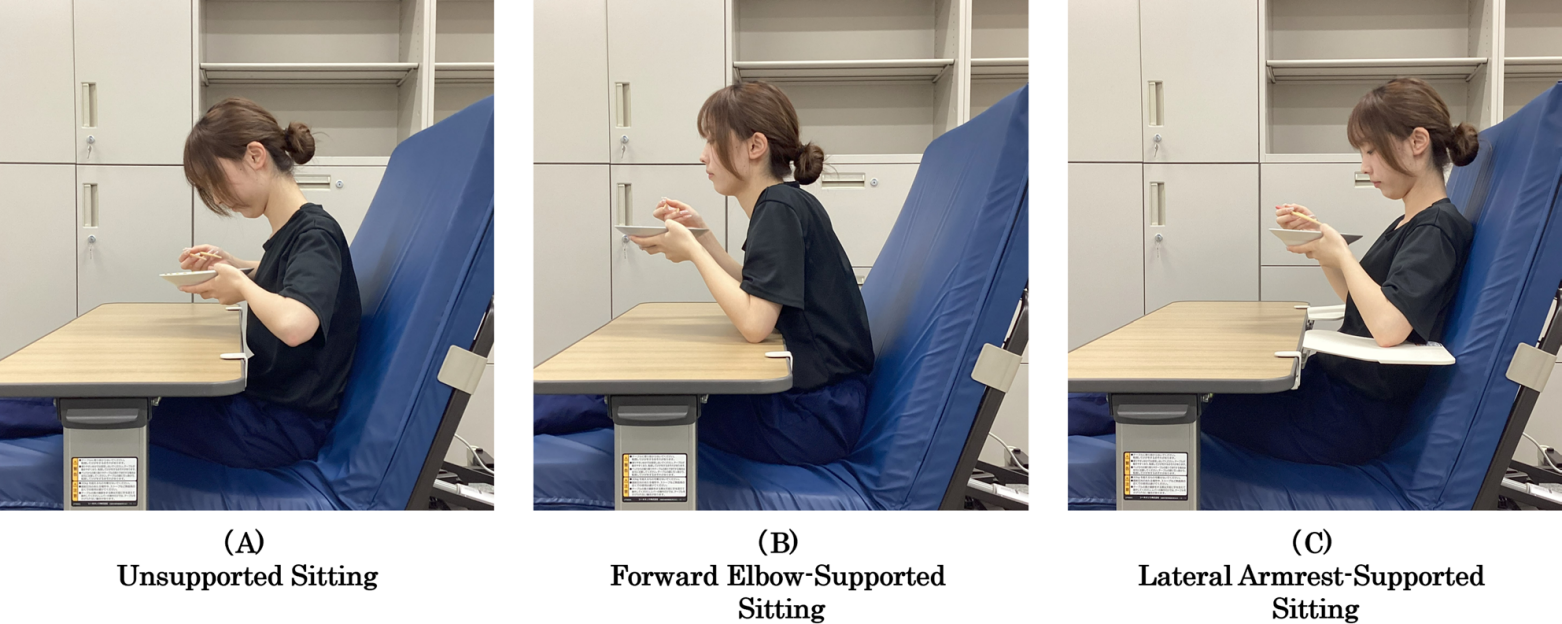
Experimental setup and seated postural conditions. Schematic illustration of the experimental setup and the three seated postural conditions evaluated during simulated eating. Condition **A**: unsupported sitting without external upper-limb support. Condition **B**: forward elbow-supported sitting using an overbed table. Condition **C**: lateral armrest-supported sitting using bilateral side-mounted armrests (Emi Table configuration). All measurements were conducted on a hospital bed with standardized backrest and knee-gatch angles

For both Conditions B and C, the table height was adjusted so that the elbow support surface was positioned approximately 3 cm above the umbilicus and 8 cm anterior to the abdominal surface, ensuring consistent upper-limb support geometry across participants. Figure 1 illustrates the experimental setup and postural configurations.

### Instrumentation and outcome measures

#### Thoracic Kyphosis index

Thoracic kyphosis was assessed using the flexicurve method between the spinous process of the seventh cervical vertebra (C7) and the spinous process of the fourth lumbar vertebra (L4). The thoracic kyphosis index was calculated using the following formula:

$$\:\text{Kyphosis index}=\left(\frac{\text{height of the }\mathrm{curve}}{\text{length of the curve}}\right)\times\:100$$

as previously described [11].

This method has been widely used as a non-radiographic approach for quantifying thoracic curvature and has demonstrated acceptable reliability and validity in previous studies [12].

### Cervical inclination angle

The cervical inclination angle was defined as the angle between:

- A line connecting the spinous process of the seventh cervical vertebra (C7) and the greater trochanter, representing trunk orientation
- A line connecting the external auditory meatus and the vertex, representing head orientation

This definition was selected to quantify head orientation relative to trunk alignment in the sagittal plane. Because the primary aim of the present study was to evaluate head–trunk interaction during simulated eating, a trunk-referenced angular definition was considered more appropriate than an angle referenced to a horizontal or vertical plane. Previous kinematic studies have emphasized that during functional upper limb tasks, evaluating the orientation of the head relative to the trunk is essential, as the cervical spine continuously compensates for trunk movements to maintain head stability [13].

Smaller cervical inclination angle values indicate greater cervical extension relative to the trunk, whereas larger values reflect a more flexed head position relative to trunk alignment. The present study did not define a normative neutral cervical reference angle; therefore, values should be interpreted as relative differences between postural conditions rather than absolute indicators of deviation from a standardized neutral posture.

#### Image-based measurement and landmark identification

Thoracic kyphosis index and cervical inclination angle were measured from lateral-view digital images using clearly defined anatomical landmarks. Prior to image acquisition, relevant anatomical landmarks (including C7 and the greater trochanter) were identified by palpation and their positions confirmed. After photography, the corresponding landmark locations were marked on the images based on the palpated reference points to enhance measurement consistency. Participants wore similar loose-fitting upper and lower garments across all conditions to reduce variability in landmark identification. Image acquisition was conducted under consistent positioning and lighting conditions to facilitate clear visualization of anatomical reference points.

### Muscle stiffness

Muscle stiffness of the upper trapezius and middle deltoid was measured on the dominant (right) side using a handheld muscle hardness meter (TDM-Z2BT; Try-All Co., Ltd., Japan). This device quantifies muscle hardness by applying a standardized mechanical indentation to the tissue surface and measuring the resistance of the underlying muscle.

The obtained value reflects the mechanical properties of superficial muscle tissue and has been used as a non-invasive indicator of muscle loading in postural tasks.

Compared with surface electromyography (EMG), which primarily evaluates electrical activation patterns, muscle hardness measurement provides a simple and objective method for assessing sustained muscular tension under quasi-static conditions [9, 10]. All participants performed the simulated eating task using the right hand, ensuring consistency in limb dominance across measurements.

The upper trapezius was selected due to its role in shoulder girdle elevation and its potential contribution to accessory respiratory activity under increased ventilatory demand. The middle deltoid was chosen to reflect sustained upper-limb stabilization load during abducted or semi-elevated arm positions required in simulated eating and elbow-supported sitting.

Measurements were obtained at standardized anatomical locations over the muscle belly (upper trapezius: midpoint between C7 and the acromion; middle deltoid: midpoint between the acromion and deltoid tuberosity) in accordance with established guidelines for non-invasive superficial muscle assessment [14]. The aim was to estimate tonic shoulder girdle loading associated with upper-limb support configuration rather than to comprehensively assess all shoulder or trunk musculature.

Each muscle was measured three times in each condition, and the mean value of the three measurements was used for analysis.

### Vital capacity

Vital capacity (VC) was measured using a spirometer (Multi-functional Spirometer HI-801, Chest, Tokyo, Japan). Participants performed two maximal respiratory maneuvers, consisting of full inspiration followed by full expiration, in each condition, and the higher value was used for analysis. Two maneuvers were conducted in accordance with spirometry standardization guidelines to ensure reliability and reproducibility. The maximal VC approach was selected to evaluate posture-related mechanical constraints on thoracic expansion under standardized effort, thereby enabling comparison across conditions independent of spontaneous tidal variability. All measurements were conducted in accordance with the American Thoracic Society/European Respiratory Society standardization guidelines for spirometry [15].

### Subjective comfort

Subjective comfort was assessed using a Numerical Rating Scale (NRS; 0–10), with higher scores indicating greater comfort. Participants also ranked the three postural conditions according to overall comfort (1 = most comfortable, 3 = least comfortable).

### Procedures

Participants were familiarized with all postural conditions before data collection. Each posture was maintained for 3 min prior to measurement to allow postural stabilization. Outcome assessments were conducted in the following standardized sequence: thoracic kyphosis index, cervical inclination angle, muscle stiffness, vital capacity, and subjective comfort. A 2-minute seated rest interval was provided between conditions to minimize fatigue and carryover effects.

### Statistical analysis

Normality of all outcome variables was assessed using the Shapiro–Wilk test. As several variables violated assumptions of normality, non-parametric statistical analyses were applied. Differences among the three postural conditions were examined using Friedman tests. Effect sizes were calculated as Kendall’s W using the chi-square statistics obtained from the Friedman tests and were interpreted as small (W ≈ 0.1), moderate (W ≈ 0.3), and large (W ≥ 0.5) effects.

When significant main effects were identified, post-hoc pairwise comparisons were performed using the Durbin–Conover test with Bonferroni correction. Statistical significance for pairwise comparisons was determined based on Bonferroni-adjusted p-values. Subjective comfort rankings were analyzed using chi-square tests. Statistical significance was set at *p* < .05.

All statistical analyses were performed using R version 4.5.2 (R Foundation for Statistical Computing, Vienna, Austria).

## Results

### Participant characteristics

Participant characteristics are summarized in Table 1. The study sample consisted of 40 healthy adults (20 males and 20 females). All participants completed all three postural conditions, and no missing data were observed.

**Table 1:** Participant characteristics

Variable	All (n = 40)	Male (n = 20)	Female (n = 20)
Age (years)	20.9 ± 1.0	20.7 ± 0.9	21.1 ± 1.1
Height (cm)	164.7 ± 8.4	170.8 ± 5.7	158.5 ± 5.8
Weight (kg)	57.3 ± 10.2	64.0 ± 9.8	50.7 ± 5.0
BMI (kg/m2)	21.1 ± 2.6	21.9 ± 2.8	20.2 ± 2.1

Participant characteristics of the study sample

Values are presented as mean ± standard deviation (SD)

*BMI* body mass index

### Postural outcomes

#### Thoracic Kyphosis index

**Fig. 2:**
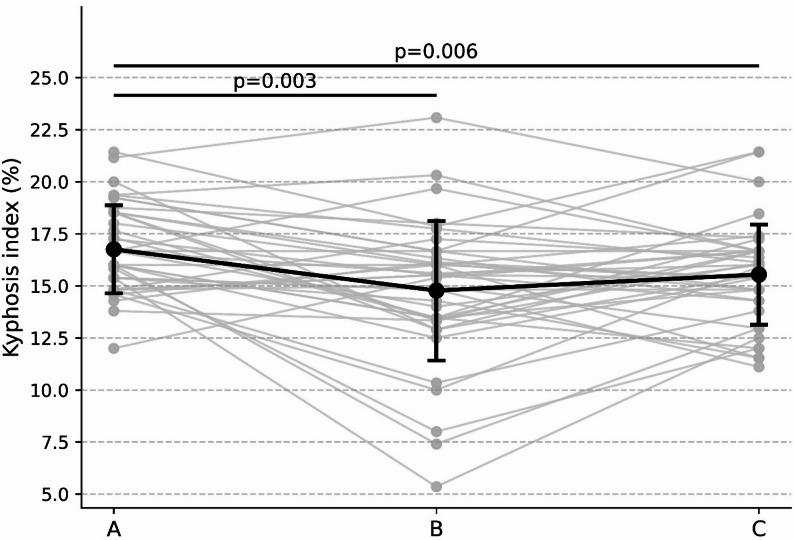
Thoracic kyphosis index across sitting conditions. Thoracic kyphosis index measured under the three sitting conditions: unsupported sitting (**A**), forward elbow-supported sitting (**B**), and lateral armrest-supported sitting (**C**). Thin lines represent individual participant trajectories, and thick lines represent group mean ± SD

Thoracic kyphosis index differed significantly across the three sitting conditions (Friedman χ²(2) = 12.68, *p* = .002; Kendall’s W = 0.16). Post-hoc pairwise comparisons using the Durbin–Conover test with Bonferroni correction indicated that forward elbow-supported sitting (condition B) resulted in a significantly lower kyphosis index than unsupported sitting (condition A) (*p* = .003). Lateral armrest-supported sitting (condition C) also demonstrated a significantly lower kyphosis index compared with condition A (*p* = .006). No significant difference was observed between conditions B and C. Individual trajectories and group mean ± SD values are shown in Fig. 2.

### Cervical inclination angle

**Fig. 3:**
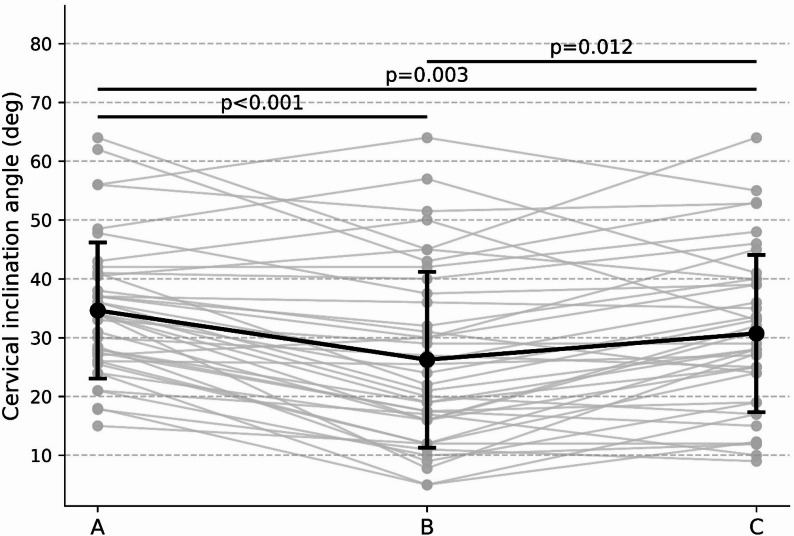
Cervical inclination angle across sitting conditions. Cervical inclination angle measured under the three sitting conditions (smaller values indicate greater cervical extension). Individual paired responses and group mean ± SD are shown for conditions **A**–**C**

Cervical inclination angle showed a marked effect of sitting condition (Friedman χ²(2) = 27.34, *p* < .001; Kendall’s W = 0.34). Post-hoc pairwise comparisons revealed that forward elbow-supported sitting (condition B) produced significantly smaller cervical inclination angle values than unsupported sitting (condition A) (*p* < .001) and lateral armrest-supported sitting (condition C) (*p* = .012). Lateral armrest-supported sitting also showed significantly smaller values than unsupported sitting (*p* = .003). These results are illustrated in Fig. 3.

### Muscle stiffness

#### Upper trapezius

**Fig. 4:**
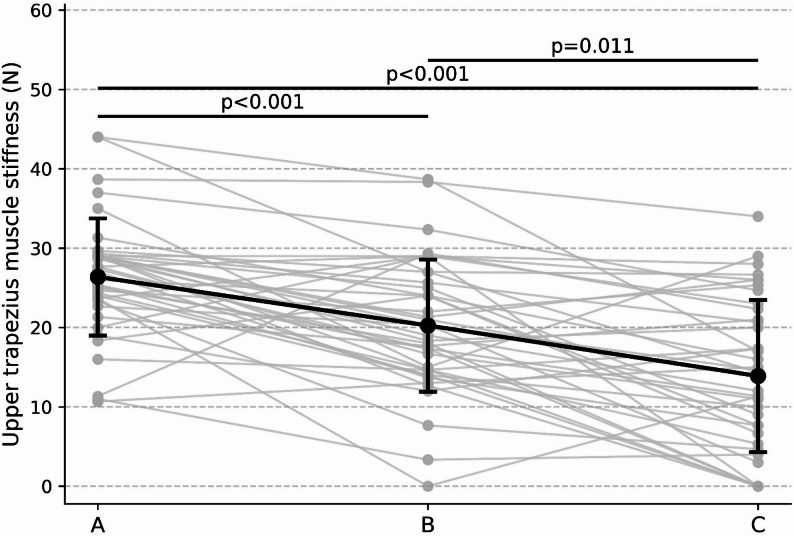
Upper trapezius muscle stiffness across sitting conditions. Upper trapezius muscle stiffness measured across the three sitting conditions. Individual trajectories are shown with superimposed group mean ± SD

A strong effect of sitting condition was observed for upper trapezius muscle stiffness (Friedman χ²(2) = 33.82, *p* < .001; Kendall’s W = 0.42). Post-hoc analyses demonstrated that lateral armrest-supported sitting (condition C) resulted in significantly lower trapezius stiffness than unsupported sitting (condition A) (*p* < .001) and forward elbow-supported sitting (condition B) (*p* = .003). In addition, condition B showed significantly lower stiffness than condition A (*p* < .001). These results are presented in Fig. 4.

### Middle deltoid

Middle deltoid muscle stiffness also differed significantly among conditions (Friedman χ²(2) = 6.78, *p* = .034; Kendall’s W = 0.09). Post-hoc comparisons indicated that stiffness was significantly lower in lateral armrest-supported sitting (condition C) than in forward elbow-supported sitting (condition B) (*p* = .039). No significant differences were observed between conditions A and B or between conditions A and C.

### Pulmonary function

#### Vital capacity

**Fig. 5:**
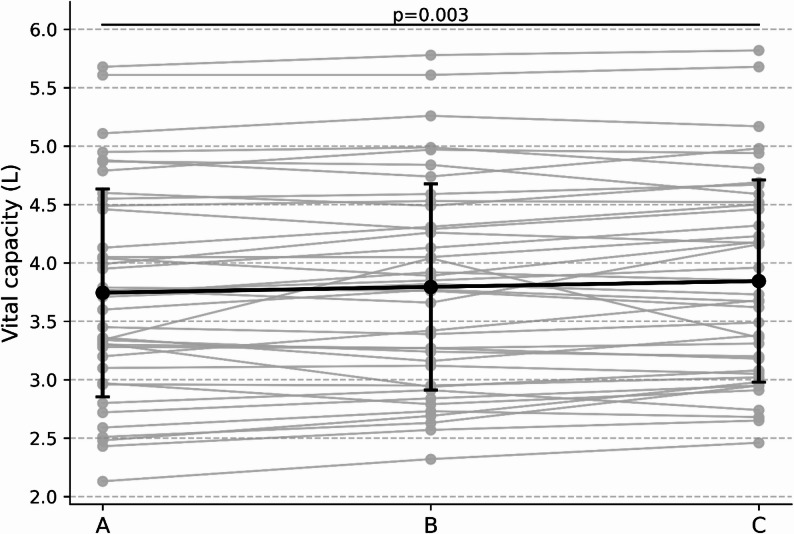
Vital capacity across sitting conditions. Vital capacity (VC) measured under the three sitting conditions. Individual participant trajectories and group mean ± SD are shown

Vital capacity (VC) differed significantly across the three sitting conditions (Friedman χ²(2) = 10.71, *p* = .005; Kendall’s W = 0.13). Post-hoc pairwise comparisons indicated that lateral armrest-supported sitting (condition C) produced significantly greater VC than unsupported sitting (condition A) (*p* = .003). VC values in forward elbow-supported sitting (condition B) did not differ significantly from those in either condition A or condition C. These results are shown in Fig. 5.

### Subjective comfort

Subjective comfort ratings differed substantially among the three conditions. Numerical Rating Scale (NRS) scores were highest in lateral armrest-supported sitting (condition C; 8.00 ± 1.48), intermediate in unsupported sitting (condition A; 5.97 ± 2.37), and lowest in forward elbow-supported sitting (condition B; 4.40 ± 1.93). Friedman testing revealed a significant main effect of sitting condition (χ²(2) = 43.78, *p* < .001; Kendall’s W = 0.55).

Post-hoc comparisons using the Durbin–Conover test with Bonferroni correction confirmed that condition C was rated significantly more comfortable than both condition A (*p* < .001) and condition B (*p* < .001), and condition A was rated significantly more comfortable than condition B (*p* < .001).

Rank-order analysis similarly demonstrated significant differences across conditions (Friedman χ²(2) = 42.65, *p* < .001; Kendall’s W = 0.53). Lateral armrest-supported sitting (condition C) was ranked as the most comfortable condition by 80% of participants, whereas forward elbow-supported sitting (condition B) was ranked as the least comfortable condition by 70%. Detailed ranking distributions are presented in Table 3, whereas descriptive and statistical results for NRS scores are summarized in Table 3.

**Table 2:** Rank-order distribution of subjective comfort across the three sitting conditions

Rank	(A) Unsupported sitting	(B) Forward elbow-supported sitting	(C) Lateral armrest-supported sitting
1st	7 (17.5%)	1 (2.5%)	32 (80.0%)
2st	22 (55.0%)	11 (27.5%)	7 (17.5%)
3st	11 (27.5%)	28 (70.0%)	1 (2.5%)

Rank-order data are presented as the number and percentage of participants selecting each condition as most comfortable

Differences in comfort rankings were analyzed using the Friedman test. NRS scores are reported separately in Table 3

**Table 3:** Postural, muscular, respiratory, and subjective outcomes across the three sitting conditions

Outcome	(A) Unsupported sitting	(B) Forward elbow-supported sitting	(C) Lateral armrest-supported sitting	Friedman χ2	p-value	Kendall’s W
Kyphosis index	16.75 ± 2.11	14.77 ± 3.35	15.54 ± 2.41	12.68	0.002	0.16
Cervical inclination angle (deg)	34.60 ± 10.8	26.4 ± 9.20	30.8 ± 10.5	27.34	< .001	0.34
Muscle stiffness (N)						
Upper trapezius	26.38 ± 7.38	20.25 ± 8.34	13.89 ± 9.59	33.82	< .001	0.42
Middle deltoid	20.81 ± 7.14	21.13 ± 5.36	17.92 ± 6.02	6.78	0.034	0.09
Vital capacity (L)	3.74 ± 0.89	3.79 ± 0.88	3.84 ± 0.87	10.71	0.005	0.13
Numerical rating scale (0–10)	5.97 ± 2.37	4.40 ± 1.93	8.00 ± 1.48	43.78	< .001	0.55

Summary of postural alignment, muscle stiffness, pulmonary function, and subjective comfort under unsupported sitting (A), forward elbow-supported sitting (B), and lateral armrest-supported sitting (C)

Values are presented as mean ± standard deviation (SD)

Group differences were analyzed using the Friedman test. Chi-square (χ²) statistics, corresponding p values, and Kendall’s W are reported as effect size

When significant main effects were identified, post-hoc pairwise comparisons were performed using the Durbin–Conover test with Bonferroni-adjusted p-values

### Outcomes summary

Group-level descriptive statistics and statistical comparisons for all postural, muscular, respiratory, and subjective outcomes are presented in Table 3.

## Discussion

### Main findings

This study is the first to quantitatively examine the biomechanical and respiratory effects of lateral armrest support during simulated eating. The principal findings were that lateral armrest-supported sitting demonstrated improved thoracic alignment and reduced shoulder girdle muscle stiffness compared with unsupported sitting, and was associated with greater vital capacity relative to unsupported sitting. Compared with forward elbow-supported sitting, lateral armrest-supported sitting resulted in more favorable cervical alignment and higher subjective comfort ratings. These findings indicate condition-specific biomechanical advantages rather than uniform superiority across all outcome measures, suggesting that the configuration and direction of upper-limb support substantially influence postural and respiratory mechanics during eating-related seated tasks.

Beyond statistical significance, the magnitude of change observed in thoracic alignment, cervical inclination angle, and vital capacity indicates meaningful biomechanical differences between postural configurations under controlled conditions. The reported effect sizes (Kendall’s W = 0.13–0.47) suggest non-trivial within-subject effects. However, the clinical relevance of these findings requires confirmation in patient populations.

### Postural mechanisms

Both forward elbow-supported sitting and lateral armrest-supported sitting were associated with reduced thoracic kyphosis compared with unsupported sitting. However, only forward elbow-supported sitting induced pronounced cervical extension, as evidenced by significantly smaller cervical inclination angle values. Excessive cervical extension has been identified as a postural factor that may disrupt the coordination between swallowing and respiration, potentially increasing aspiration risk during eating tasks [16].

The marked cervical extension observed in the forward elbow-supported condition likely reflects a compensatory strategy to maintain forward gaze during trunk flexion rather than a desirable effect of elbow support itself. In contrast, lateral armrest support allowed participants to unload the upper limbs without adopting a forward-leaning posture, thereby maintaining a more neutral cervical alignment. This observation is consistent with previous reports indicating that upright or neutral seated postures minimize cervical compensations and optimize global spinal alignment [17]. Importantly, the present findings suggest that the direction of upper-limb support, rather than the mere presence of support, plays a critical role in shaping spinal alignment during seated eating.

### Reduction in accessory respiratory muscle load

Lateral armrest-supported sitting was associated with a substantial reduction in upper trapezius muscle stiffness and a smaller but significant reduction in middle deltoid stiffness. The upper trapezius may function as an accessory respiratory muscle under increased ventilatory demand [18]; however, in the present static sitting condition, muscle stiffness should primarily be interpreted as reflecting postural and upper-limb stabilization demand rather than direct respiratory muscle activity.

The middle deltoid does not directly contribute to respiration but was included to estimate sustained upper-limb stabilization load associated with different support configurations. The observed reductions in trapezius and deltoid stiffness therefore suggest decreased shoulder girdle loading associated with lateral upper-limb support. While other muscles such as the anterior deltoid or latissimus dorsi may contribute under dynamic or load-bearing conditions, the present measurements focused on muscles most directly involved in sustained upper-limb stabilization in the tested postural configurations.

Although muscle stiffness provides only an indirect estimate of neuromuscular activity, these findings indicate reduced postural muscular demand rather than definitive changes in respiratory muscle recruitment.

### Ventilatory consequences

Vital capacity was significantly greater in the lateral armrest-supported condition than in unsupported sitting, indicating more favorable ventilatory mechanics. Previous studies have demonstrated that upright posture and reduced spinal flexion improve respiratory muscle strength and lung volumes by optimizing diaphragm length–tension relationships and chest wall compliance [4].

In the present study, the combination of improved thoracic alignment, reduced cervical extension, and decreased shoulder girdle loading may have facilitated more effective thoracoabdominal expansion. These biomechanical changes are consistent with established principles of respiratory physiology. Although maximal VC reflects mechanical capacity rather than spontaneous ventilation during eating, it enables isolation of posture-related thoracic constraints under standardized effort. Future studies incorporating tidal breathing analysis during sustained eating posture may provide complementary insight into real-world respiratory demand.

### Comparison with previous literature

Anterior elbow support has long been recommended in clinical practice as a means of unloading the upper limbs during sitting; however, its combined postural and respiratory consequences have not been systematically quantified. The present findings clarify that while forward elbow support can reduce thoracic kyphosis, it simultaneously induces greater cervical extension, a postural pattern that may be unfavorable during eating tasks.

To our knowledge, no previous studies have evaluated lateral armrest support as an alternative upper-limb support strategy during seated eating. By integrating objective measures of postural alignment, cervical inclination angle, muscle stiffness, pulmonary function, and subjective comfort, the present study provides a multidimensional assessment that addresses an important gap in the literature on eating-related seating biomechanics.

### Clinical implications

Although these findings provide preliminary biomechanical insight, they were obtained exclusively in healthy young adults. Therefore, direct clinical application should be approached with caution. Patient-based validation studies are necessary to determine whether similar postural and respiratory responses occur in individuals with respiratory disease, neuromuscular weakness, or reduced endurance during meals. Such investigations will be essential before clinical recommendations can be formally established. This approach may be particularly relevant in bed-based or long-sitting contexts, where postural options are constrained by environmental, medical, or staffing factors.

From a rehabilitation engineering perspective, the results provide foundational evidence to inform the design of assistive seating systems that support both postural stability and respiratory efficiency. Importantly, the intervention examined in this study is simple, low-cost, and readily implementable, suggesting potential for broad applicability in clinical and care settings.

### Limitations

Several limitations should be acknowledged. This study was conducted exclusively in healthy young adults under short-term experimental conditions. Therefore, direct extrapolation of these findings to older adults or clinical populations with impaired ventilatory reserve, balance dysfunction, or neuromuscular weakness should be considered premature, and patient-based validation is required before clinical application.

Measurements were obtained during simulated rather than actual food intake and did not capture fatigue-related changes during prolonged meals. Muscle stiffness was used as an indirect indicator of muscular load; direct measures such as surface electromyography would provide complementary insight. In addition, although palpation-based landmark identification and consistent imaging procedures were employed to reduce measurement error, formal intra- and inter-rater reliability testing was not performed. Future studies should incorporate reliability assessments to further strengthen methodological rigor.

Although task configuration was standardized across conditions, minor anthropometric differences among participants may have influenced shoulder positioning, particularly in the forward elbow-supported condition. Future studies may consider adjusting table height relative to individual acromial or elbow height to further minimize such variability.

In addition, the present study did not assess center-of-pressure (COP) or pressure distribution, which may provide further insight into postural stability and task-related demand. Future investigations incorporating pressure mapping or COP analysis could help clarify the suitability of these seating configurations for individuals with impaired balance capacity.

### Future directions

Future studies should evaluate the effects of lateral armrest support in clinical populations with respiratory or neuromuscular disorders. Incorporating direct measures of respiratory effort, such as surface electromyography or respiratory inductance plethysmography, may help clarify underlying neuromuscular mechanisms. In addition, long-term outcomes—including dyspnea during meals, swallowing safety, and nutritional efficiency—should be examined. Further optimization of armrest height, angle, and configuration based on individual characteristics may also enhance clinical utility.

## Conclusions

Lateral armrest support during simulated seated eating was associated with improved spinal alignment, reduced cervical extension (as indicated by cervical inclination angle), decreased shoulder girdle muscular demand, and greater ventilatory capacity relative to unsupported sitting in healthy young adults. These findings provide preliminary biomechanical evidence under controlled experimental conditions. Further research is required to determine the clinical relevance and applicability of this seating configuration in populations with respiratory or neuromuscular impairment.

## Data Availability

The datasets generated and analyzed during the current study are available from the corresponding author upon reasonable request.
